# Grazing resistance and poor food quality of a widespread mixotroph impair zooplankton secondary production

**DOI:** 10.1007/s00442-020-04677-x

**Published:** 2020-06-05

**Authors:** Csaba F. Vad, Claudia Schneider, Dunja Lukić, Zsófia Horváth, Martin J. Kainz, Herwig Stibor, Robert Ptacnik

**Affiliations:** 1 WasserCluster Lunz, Dr. Carl Kupelwieser Promenade 5, 3293 Lunz am See, Austria; 2 Balaton Limnological Institute, Centre for Ecological Research, Klebelsberg Kuno u. 3, 8237 Tihany, Hungary; 3 Department of Limnology and Bio-Oceanography, University of Vienna, Althanstraße 14, 1090 Vienna, Austria; 4 Department of Biology II, Ludwig-Maximilians-University Munich, Großhaderner Str. 2, 82152 Planegg-Martinsried, Germany

**Keywords:** Mixotrophic chrysophytes, Food web, Nutritional value, *Dinobryon*, Zooplankton

## Abstract

Growing evidence suggests that global climate change promotes the dominance of mixotrophic algae especially in oligotrophic aquatic ecosystems. While theory predicts that mixotrophy increases trophic transfer efficiency in aquatic food webs, deleterious effects of some mixotrophs on consumers have also been reported. Here, using a widespread mixotrophic algal genus *Dinobryon*, we aimed to quantify how colonial taxa contribute to secondary production in lakes. We, therefore, studied the dietary effects of *Dinobryon divergens* on Cladocera (*Daphnia longispina*) and Copepoda (*Eudiaptomus gracilis*), representing two main taxonomic and functional groups of zooplankton. In feeding experiments, we showed that *Dinobryon* was largely grazing resistant and even inhibited the uptake of the high-quality reference food in *Daphnia. Eudiaptomus* could to some extent compensate with selective feeding, but a negative long-term food quality effect was also evident. Besides, *Eudiaptomus* was more sensitive to the pure diet of *Dinobryon* than *Daphnia*. Low lipid content and high C:P elemental ratio further supported the low nutritional value of the mixotroph. In a stable isotope approach analysing a natural plankton community, we found further evidence that carbon of *Dinobryon* was not conveyed efficiently to zooplankton. Our results show that the increasing dominance of colonial mixotrophs can result in reduced dietary energy transfer to consumers at higher trophic levels. In a wider perspective, global climate change favours the dominance of some detrimental mixotrophic algae which may constrain pelagic trophic transfer efficiency in oligotrophic systems, similarly to cyanobacteria in eutrophic lakes.

**Electronic supplementary material:**

The online version of this article (10.1007/s00442-020-04677-x) contains supplementary material, which is available to authorized users.

## Introduction

Given the key importance of plankton communities for aquatic biomass production and biogeochemical cycling, understanding their global change-mediated shifts is ecologically highly relevant. Changes in plankton of lakes undergoing eutrophication is widely discussed in the context of climate change, with a strong focus on the increased dominance of cyanobacteria (O’Neil et al. 2012; Taranu et al. 2015; Huisman et al. 2018). Much less attention is paid to the shifts in less productive (oligo- to mesotrophic) lakes, which are typically less impacted by humans and are highly valuable for recreation and fishing. Climate change has wide-ranging consequences on the plankton of these systems, driving compositional shifts (Winder et al. 2009; Forsström et al. 2013), with implications for ecosystem functioning and the provision of ecosystem services (e.g., algal blooms, Callieri et al. 2014). Furthermore, oligotrophication of freshwaters became a global trend (Jeppesen et al. 2005; Tong et al. 2017), and its interactive effects with climate change has only recently started to gain considerable interest (Verbeek et al. 2018; Cabrerizo et al. 2020). This calls for a need to better understand climate-change driven planktonic changes in nutrient-poor lakes. Special attention needs to be paid to the functional role of key taxa which possess traits allowing them to dominate in these plankton communities.

There are some particular algal traits that are favourable under nutrient-poor conditions, such as mixotrophy (i.e., combining phototrophy and phagotrophy). Mixotrophic algae are able to acquire limiting nutrients via bacterivory (Katechakis and Stibor 2006; Fischer et al. 2017), which provides them a competitive advantage over obligate autotrophs and allows them to dominate especially in oligotrophic and stratifying systems (Domaizon et al. 2003; Zubkov and Tarran 2008; Hartmann et al. 2012). As climate warming is predicted to result in extended periods of thermal stratification and consequently in epilimnetic nutrient depletion in lakes (Adrian et al. 2009), it can be expected that the contribution of mixotrophs to total phytoplankton biomass will also increase in their favoured habitats. Besides warming, mixotrophic algae also benefit from higher bacterial production under increased availability of dissolved organic carbon (DOC; Bergström et al. 2003; Forsström et al. 2013; Wilken et al. 2018). A gradual increase in DOC was observed in many temperate lakes and is predicted to accelerate under current climate-change scenarios (Bergström et al. 2003; Roulet and Moore 2006; de Wit et al. 2016). Global climate change can, therefore, be expected to enhance the dominance of mixotrophic algae in nutrient-poor systems. While an increasing number of studies attempt to quantify their roles in the microbial loop as bacterivores (Domaizon et al. 2003; Hartmann et al. 2012; Ptacnik et al. 2016), the consequences of their dominance for higher trophic levels remain largely unexplored. Recent research has suggested that mixotrophic protists may enhance secondary production due to their less variable stoichiometric elemental ratios compared to autotrophs (Katechakis et al. 2005; Moorthi et al. 2017). Moreover, by bypassing the microbial loop (Ptacnik et al. 2016), mixotrophy is expected to increase trophic transfer efficiency from bacteria to higher trophic levels (Ward and Follows 2016). At the same time, toxic effects were reported in a wide range of mixotrophs, many of them being important components of harmful algal blooms (Watson et al. 2015; Flynn et al. 2018).

In freshwater ecosystems, chrysophytes (Chrysophyceae) represent a major mixotrophic group accounting for a considerable share of phytoplankton in oligo- and mesotrophic lakes that also frequently form blooms (Watson et al. 1997; Ptacnik et al. 2008). Their expected increase in dominance urges for a better understanding about their role in pelagic trophic relationships, such as their contribution for secondary production, especially as some species are potentially toxin-producing (Boenigk and Stadler 2004; Hiltunen et al. 2012; Watson et al. 2015). We particularly lack information on the bottom-up role of colonial taxa among genera like *Uroglenopsis* and *Dinobryon*. *Dinobryon* comprises many common species that may form large colonies, and is very widespread in lakes of the temperate zone (Lehman 1976; Sandgren 1988; Reynolds et al. 2002). It is a key component in pelagic carbon cycling given its ability to dominate phytoplankton biomass (Dokulil and Skolaut, 1991; Pugnetti and Bettinetti, 1999; Urrutia‐Cordero et al., 2017) and its role as a major bacterial grazer (Bird and Kalff 1986; Sanders et al. 1989; Domaizon et al. 2003). However, there is a long-standing debate on its dietary value for zooplankton, and therefore the potential implications of *Dinobryon* dominance for pelagic trophic transfer efficiency are largely unknown. It is commonly assumed to be grazing resistant due to its large colonies, in which cells are individually surrounded by a vase-like lorica, but with a lack of quantitative evidence (Müller-Navarra and Lampert 1996; Agrawal 1998; Colina et al. 2016). Knisely and Geller (1986) reported that *Dinobryon* was largely avoided by zooplankton when fed with a diverse natural phytoplankton community, but the exact mechanism of selectivity was not investigated. Negative effects of *Dinobryon* dominance on zooplankton secondary production are reported based on correlative evidence from mesocosm (Faithfull et al. 2011) and field studies (Talling 2003). At the same time, *Dinobryon* was repeatedly found to proliferate in situations of low crustacean zooplankton biomass, thereby indicating sensitivity to grazing (Svensson and Stenson 1991; Fussmann 1996; Sommer et al. 2003) and empty loricas found in *Daphnia* guts provided evidence for ingestion (Infante 1973). Some authors even considered *Dinobryon* spp. being among the primary food sources of zooplankton in some freshwater systems (Bertoni et al. 2002; Jäger et al. 2014). Therefore, as *Dinobryon* represents arguably the most widespread taxon of colonial mixotrophic algae in freshwater lakes, it is ecologically important to clarify its dietary effects on different zooplankton taxa.

The main taxa of crustacean zooplankton, Cladocera and Copepoda, differ considerably in their nutritional demands and feeding modes. Most cladocerans (incl. *Daphnia*) have very limited ability to feed selectively. In contrast, copepods can switch between alternative feeding modes depending on the food type, and are capable of a fine-scale discrimination and active uptake of food particles based on size, motility and chemical cues (DeMott 1986; Kerfoot and Kirk 1991; Atkinson 1995; Kiørboe 2011). Copepods (including calanoids) are able to exploit a wider range of particles, showing a preference for the larger fraction of phytoplankton (Sommer and Sommer 2006) and are known to feed on even larger prey items such as ciliates and rotifers (Kleppel 1993; Adrian and Schneider-Olt 1999; Brandl 2005). It follows that cladocerans, known to feed efficiently only on smaller particles (e.g., in most *Daphnia* species the upper limit is ~ 30–40 µm; Burns 1968; Geller and Müller 1981), are more sensitive to mechanic interference of their filtration apparatus by larger algae. In terms of food quality, cladocerans are generally more prone to phosphorus-limitation, whereas copepods are more nitrogen-demanding, especially in their later developmental stages (Hessen 1992; Meunier et al. 2016; Branco et al. 2018). The essential fatty acid composition of these major groups of crustacean zooplankters also reflects distinct nutritional demands (Persson and Vrede 2006; Smyntek et al. 2008). These overall imply different behavioural and physiological responses to chrysophyte and specifically to *Dinobryon* diet, which merits further investigation given the expected increase in dominance of this algal group in lakes.

Our main goal here is to quantify the effects of colonial mixotrophs on crustacean zooplankton secondary production by using the widespread *Dinobryon* as a model organism. Specifically, we aim to reveal its degree of grazing resistance and nutritional value for representative crustacean zooplankton taxa. To this end, we experimentally test in laboratory feeding experiments how *Dinobryon divergens* affects (1) ingestion rates and (2) survival and reproduction of common lake zooplankters, i.e., the cladoceran *Daphnia longispina* and the copepod *Eudiaptomus gracilis*. For detailed understanding of the observed dietary effects, nutritional quality of *D. divergens* is assessed by analysing its fatty acid and elemental composition. Finally, we aim to quantify how much *Dinobryon* contributes to secondary production in natural lake plankton based on the analysis of carbon and nitrogen stable isotope ratios.

## Materials and methods

### Cultivation of phyto- and zooplankton

*Dinobryon divergens* O. E. Imhof was isolated from Lake Lunz, an oligotrophic (5–8 μg total phosphorus L^−1^) lake in the montane region (608 m a.s.l) of the Eastern Alps (47°51.2′N 15°3.1′E; for more details on the lake, see e.g., Kainz et al. 2017). It was cultivated in a medium based mostly on sterile-filtered (0.2 µm pore size) water from the lake (90% of the total volume) which was enriched with WEES medium (Kies 1967) without soil extract. This medium proved to be the most suitable for cultivation of an array of chrysophyte species isolated from Lake Lunz in previous bioassays (data not shown). *Cryptomonas* sp. (SAG 26.80) was grown in a slightly modified medium (consisting of 80% sterile-filtered lake water and 20% WEES medium) given its insufficient growth in the medium with higher lake water content. Cultures were kept in batch cultures in a walk-in chamber at 18 °C under a constant 16:8 light:dark cycle (illumination: ~ 170 µmol photon m^−2^ s^−1^), and were regularly diluted to keep them in exponential growth phase.

The cladoceran *Daphnia cf. longispina* (species belonging to the *D. longispina* complex, hereinafter referred to as *D. longispina*) and the copepod *E. gracilis* (G.O. Sars) were also isolated from Lake Lunz. Stock cultures (a clonal line in the case of *D. longispina*) of zooplankters were kept in pre-filtered (0.2 µm pore size) lake water under the above-mentioned conditions for several weeks before the experiments and were fed with *Cryptomonas* sp. (cell length: 10–17 µm, diameter: 5–9 µm).

### Grazing and life-history experiments

We compared ingestion rates on *D. divergens* to the naked flagellate *Cryptomonas* sp. in grazing experiments. Cryptophytes are generally seen as a good food for crustacean zooplankton and *Cryptomonas* sp. was therefore chosen as a reference diet (Ahlgren et al. 1990; von Elert and Stampfl 2000). General features of the two algae are presented in Table 1. We used a cohort of ovigerous *E. gracilis* females (~ 3-weeks-old), which were raised from eggs in the lab and were grown on *Cryptomonas* under food-saturated conditions (≥ 1 mg carbon L^−1^). In *D. longispina*, offspring of the second clutch of genetically identical and synchronized females were raised under the same conditions and were used (~ 3-weeks-old). In the experiment, *D. divergens* and *Cryptomonas* were provided as sole food as well as in three different mixtures (biomass ratio: 0.75, 0.5, 0.25). This design translated into five different treatment incubations, each with the same initial food concentration, which corresponded to 1 mg C L^−l^ calculated from cell biovolumes with the conversion factor by Rocha and Duncan (1985). Biovolumes of the two algal species were quantified separately by measuring axial dimensions of 30 cells in both cultures, using the formula for ‘prolate spheroid’. In *D. divergens*, the determination of biovolume was based only on cells without loricas. In each of the experimental vials, we incubated four animals in 40 mL of filtered (0.2 µm pore size) lake water enriched with algae. All treatments were replicated three times. The experiments were run for 8 h under constant light and temperature (18 °C) and were gently mixed in every 2 h to keep the algae in a homogenous suspension. Cell densities were quantified by flow cytometric analysis (Beckman CytoFLEX) and prey-specific ingestion rates were calculated following Frost (1972).

**Table 1:** Features of the cultivated *Dinobryon divergens* and *Cryptomonas* sp. used in the grazing experiments

	*D. divergens*	*Cryptomonas* sp.
Average cell biovolume (µm^3^)	287.1 ± 74.0	400.7 ± 109.8
Average length of individual loricas (µm)	35.9 ± 4.5	–
Average width of individual loricas (µm)	9.5 ± 1.5	–
Average length of 2-cell colonies incl. loricas (µm)	66.0 ± 3.3	–
Average length of 3-cell colonies incl. loricas (µm)	96.4 ± 4.5	–
% of naked (without lorica) single cells of all cells	12.9	–
% of single cells with lorica of all cells	17.6	–
Average colony size excluding empty loricas (no. of all cells/sum of all single cells and colonies)	1.9 ± 1.4	–
Max. number of cells per colony	12	
Average colony size including empty loricas (no. of all loricas/sum of all single loricas and those in colonies)	2.1 ± 1.6	–
% of empty loricas/all loricas	27.7	–

Biovolume and length (± SD) values are based on measurements of 30 cells in the cultures, while other features on inspecting > 600 cells or colonies

To test for long-term dietary responses of zooplankton, we monitored their survival and reproduction in feeding experiments lasting for 21 days. The three treatments here were represented by monospecific diets of the two algal cultures, *D. divergens* and *Cryptomonas*, as well as their 1:1 mixture (based on biomass), provided at saturating concentrations (≥ 1 mg C L^−1^). A cohort of *E. gracilis* females raised from eggs in the lab on *Cryptomonas* were isolated by the time they started to carry eggs (~ 3-weeks-old) and pre-incubated under the experimental conditions for 2 days until they dropped their egg sacs. This procedure allowed starting the experiment with females in similar conditions judging by the number of eggs in their first clutch (specimens with 8–10 eggs were picked). In *D. longispina*, individuals from the second clutch of females of a single clone raised on *Cryptomonas* were used. They were picked as adult, egg-carrying females (~ 2-weeks-old and carrying their second clutch) to pre-incubate them under the experimental conditions like we did with *E. gracilis*, and started the experiments by the time they released their juveniles (i.e., which was still a result of the pre-feeding on *Cryptomonas*). In the experiment, *D. longispina* females were placed individually in glass vials containing 30 mL 0.2-µm-filtered lake water enriched with algae. This design was replicated 10 times per treatment. The same setup was used for *E. gracilis* except that females were incubated together with a male specimen to allow for re-mating during the experiment necessary for clutch production (Berger and Maier 2001). Males were replaced every other day to ensure that treatment-specific effects arise exclusively from differences in females. Animals were transferred to fresh medium every other day to maintain constant and saturating food conditions (algae cell concentrations were also monitored daily in three random replicates per treatments). The experiments were run at a constant temperature (18 °C) and 16:8 h light:dark cycle. We recorded survival and reproduction of the animals daily (number of juveniles, number and size of broods).

### Analyses of food quality

During the long-term feeding experiments, we took samples for elemental and biochemical analyses (stoichiometry and fatty acid composition) from the algal food cultures. Material was retained on pre-combusted and acid-washed glass microfibre filters (Whatman GF/F).

The analyses of lipids and their fatty acids followed the methods described in detail by Heissenberger et al. (2010). In brief, lipids were extracted from freeze-dried, homogenized samples with chloroform–methanol mixture, fatty acids were derivatized to methyl esters using H_2_SO_4_‐methanol. Fatty acid methyl esters (FAME) were dried under N_2_ and redissolved in hexane before analysed by a gas chromatograph (Thermo Scientific TRACE GC Ultra equipped with a flame ionization detector) and separated with a Supelco™ SP-2560 column. FAME were identified using known standards. In addition, total lipids were quantified gravimetrically as mass fractions (mg lipids/g dry weight). All analyses were done on triplicate samples per each algal species.

Particulate organic carbon and nitrogen determination was performed by an elemental analyser (Elementar vario MICRO cube™), while particulate phosphorus was measured spectrophotometrically using the ascorbic acid colorimetric method (Hansen and Koroleff 2007) after persulphate digestion (Clesceri et al. 1999).

### Stable isotope analysis

On 25-April-2017, we collected plankton samples from Lake Lunz, during a period when *D. divergens* was a dominant member of its phytoplankton with a biomass peak above the thermocline. The first set was collected with vertical hauls with a 100 µm-mesh plankton net and *D. divergens* was sorted from the live samples with a pipette under a stereo microscope in the lab (given its dominance in this fraction of the phytoplankton it was possible to collect a sufficient amount of biomass for the analyses). Then the material was retained on Whatman GF/F filters and frozen at − 80 °C until further processing. The same was done to collect microzooplankton (ciliates, rotifers, and copepod nauplii) making use of their positive phototactic behaviour. Larger crustacean zooplankton was also collected with the 100 µm-mesh net. Multiple taxa (the cladocerans *D. longispina*, *Bosmina longispina* and the copepods *E. gracilis* and *Cyclops abyssorum*) were separated by species in the laboratory under a stereo microscope. In the two species of cladocerans, we pooled all developmental stages per species together, while for copepods we only used adults and larger copepodites. Isolated specimens of zooplankton were rinsed with distilled water and kept frozen at − 80 °C.

For seston samples, a depth-integrated water sample (20 L) was first filtered through a 100-µm sieve to remove crustacean zooplankton and then two types of seston samples were collected from the filtrate. For a small-sized fraction representing the most accessible part for cladoceran zooplankton, water was gently poured through a 20-µm sieve (to remove *Dinobryon* and other larger or colonial algae) and then the material in the filtrate was retained on Whatman GF/F filters for further analysis. For the larger-sized fraction we used the part that was retained on the 20-µm sieve. This (i.e., the 20–100-µm fraction) was, however, later omitted from the analyses as the retained amount of material was insufficient for the analysis.

Freeze-dried material of seston (0.6–0.7 mg), *D. divergens* (1.8–2.0 mg), microzooplankton (0.1–0.2 mg) and the four species of crustacean zooplankton (0.4–0.6 mg, a bulk sample of whole animals were used) were eventually analysed in three replicates using an elemental analysis—isotope ratio mass spectrometer (EA-IRMS; EA—Thermo Scientific™ FLASH 2000 HT™; IRMS—Thermo Scientific™ Delta V™ Advantage). δ^13^C and δ^15^N are reported relative to international standards (VPDB for carbon and atmospheric nitrogen for nitrogen).

### Data analyses

In the process of grazing rate estimation, negative ingestion rates were set to zero prior to the regression analyses (Nejstgaard et al. 2001). To analyse how biomass ingestion rate changes with the increasing share of *D. divergens* in the algae mixtures, we fitted both linear models and generalized additive models with smooth terms (GAMs with the ‘mgcv’ package of R; Wood 2017) on the total biomass ingested (i.e., summarized for the two algae) across treatments. Model selection was then based on Akaike’s Information Criterion (AIC), where the GAMs were generally found to be better over the linear models. We fitted separate models for *E. gracilis* and *D. longispina*.

In the next step, we tested how the increasing share of *D. divergens* influenced the ingestion rates on the reference food *Cryptomonas* with one-way ANOVA. The assumptions of normality of residuals and homogeneity of variances were tested with Shapiro–Wilk and Levene’s tests. We expected that the cladoceran will be especially sensitive to the interference with *D. divergens* colonies resulting in decreasing ingestion rates on *Cryptomonas* in food mixtures compared to that on pure diet. These effects should be smaller in *E. gracilis* due to its better ability to handle *D. divergens* or to actively select for the reference food. To test whether the ratio of ingested *D. divergens* per total ingested biomass differ between the two zooplankton species, which may indicate differences in handling abilities, we performed a two-way ANOVA with ‘species’ (*D. longispina* or *E. gracilis*) and ‘treatment’ (25, 50, 75% *D. divergens* in the food mixture) as categorical variables. Finally, for a further evidence for possible preferential feeding or feeding inhibition, we compared the relative grazing rates on *Cryptomonas* observed in the mixed-food treatments (relative to the grazing rate on pure *Cryptomonas* diet) to a calculated mean grazing rate representing no selectivity. This comparison was done by verifying overlaps in the 95% confidence intervals.

Treatment-specific differences in survival and reproduction (cumulative number of juveniles, number of broods, and mean brood size per female) throughout the long-term feeding experiment were tested with one-way ANOVA, or non-parametric Kruskal–Wallis test when a variable did not meet the assumptions of a parametric test even after data transformations (square root or log transformation; Supplementary Information, Table S1). To reveal which treatments were significantly (*p* < 0.05) different from each other, we applied post hoc Tukey HSD tests (after ANOVA) or Dunn’s multiple comparison tests (after Kruskal–Wallis test). In *D. longispina*, we additionally observed temporal differences in reproduction among the treatments. To quantify these, we performed separate one-way ANOVAs or Kruskal–Wallis tests (depending on the normality of residuals) for each day.

Stable isotope analysis revealed that the small-sized fraction of seston (one of the possible food sources) was enriched in ^13^C relative to zooplankton in our study. This is a common phenomenon in lake seston when collected similarly to our samples (i.e., as bulk), and is mostly attributed to a mixed signal of autochthonous and allochthonous particulate organic matter in the samples (Grey and Jones 1999). Taking this into account, we did not run stable isotope mixing models to compare the share of the different food types in the diet of zooplankton species. We instead compared the δ^13^C signals of small-sized seston and *D. divergens* to zooplankton by assuming a pure diet and applying literature-based enrichment factors (multiple mean ± SD fractionation values: 0.4 ± 1.3‰, Post 2002; 0.3 ± 1.28‰, McCutchan et al. 2003).

## Results

**Fig. 1:**
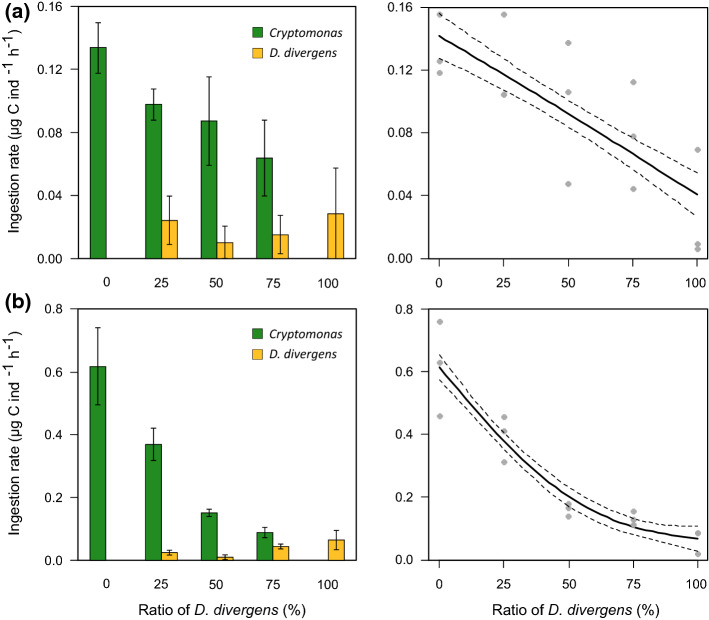
Biomass (carbon) ingestion rates (mean ± SD, *N* = 3 per treatment) of **a***Eudiaptomus gracilis* and **b***Daphnia longispina* on *Cryptomonas* sp. and *Dinobryon divergens* separately (left) and the total ingested biomass (right; dashed lines denote the standard error of the predictions) on algae mixtures with increasing ratio of *D. divergens* (generalized additive model for *E. gracilis* ingestion rate: adj. *R*^*2*^ = 0.57, approximate significance of smooth term *p* < 0.001, *N* = 15; for *D. longispina*: adj. *R*^*2*^ = 0.88, *p* < 0.001, *N* = 15)

**Fig. 2:**
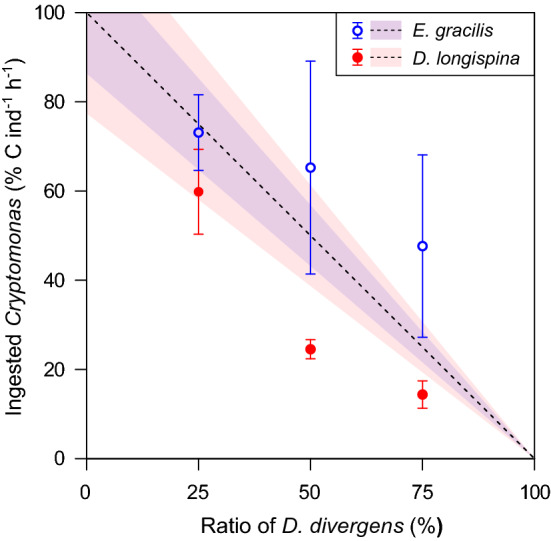
Ingestion rate on *Cryptomonas* sp. (mean ± 95% confidence interval, *N* = 3 per treatment for both zooplankton species) relative to the unialgal *Cryptomonas* treatment in the three experimental treatments with mixed food (i.e., increasing ratio of *Dinobryon divergens*). The dashed 1:1 line indicates a theoretical mean grazing rate with no feeding selectivity or feeding inhibition, with coloured bands representing the 95% confidence interval calculated from the ingestion rates on unialgal *Cryptomonas* diet

Ingestion rates were generally higher on *Cryptomonas* (reference food) for both zooplankton taxa and decreased with increasing shares of *D. divergens* (Fig. 1). In *E. gracilis*, the drop in total ingested biomass across the treatments was close to linear (Fig. 1a). At the same time, ingestion rates on *Cryptomonas* were not significantly different from what we observed on the pure diet of the same alga except for the treatment with 75% *D. divergens* (Table 2). The drop in total ingested biomass was more pronounced in *D. longispina* than in *E. gracilis* and compared to an exponential decay pattern (Fig. 1b). Ingestion rates varied on average by a factor of ~ 4–5 (0.13–0.03) in *E. gracilis*, and by a factor of ~ 10 in *D. longispina* (0.62–0.06). *Cryptomonas* ingestion rates were also decreasing with significant differences among the treatments with 0, 25 and 50% of *D. divergens* (Table 2). We did not find significant differences in the ratio of ingested *D. divergens* in the food among the two zooplankters (Supplementary Information, Fig S1 and Table S2). The grazing rates on *Cryptomonas* relative to when providing it as pure diet again showed pronounced differences among the two zooplankters as *E. gracilis* generally ingested higher biomasses of *Cryptomonas* than *D. longispina*. Besides, from 50% or more percentage of *D. divergens*, *E. gracilis* showed a trend to ingest more from the reference food compared to what can be expected based purely on the ratio of the two algae and with non-preferential grazing (although confidence intervals were overlapping between the observed and calculated data; Fig. 2). *D. longispina* responded differently by ingesting less biomass than expected (no overlaps in the confidence intervals; Fig. 2).

**Table 2:** Results of one-way ANOVA testing for treatment-specific differences in the ingestion rates on the reference food *Cryptomonas*

	*df*	*F*	*p*	Treatments
*Eudiaptomus gracilis*	3	3.90	0.055	0% = 25% = 50% > 75%
*Daphnia longispina*	3	25.11	< 0.001	0% > 25% > 50% = 75%

Treatments (increasing share of *Dinobryon divergens*) are ordered based on post hoc Tukey’s HSD tests (‘ = ’ stands for non-significant, while ‘ > ’ for significant differences)

**Fig. 3:**
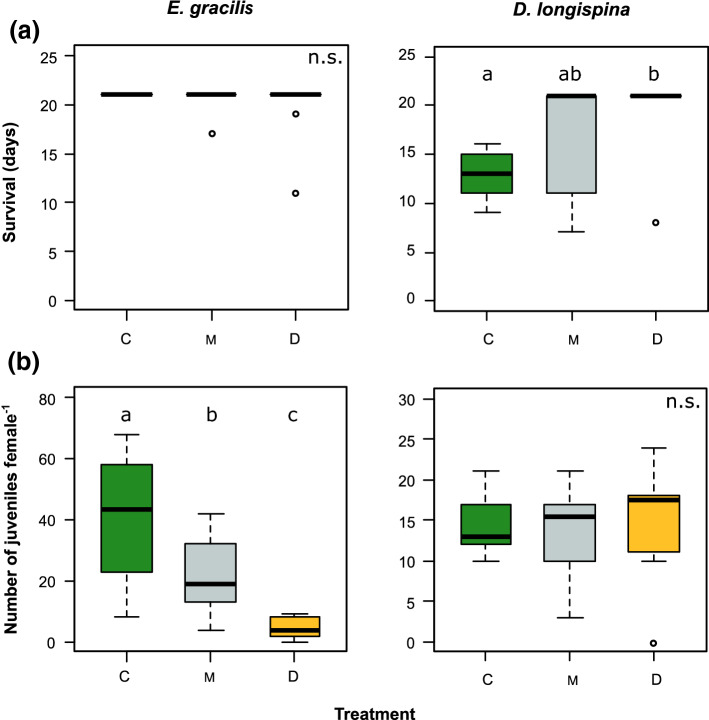
Survival on the different food types **a** and cumulative number of juveniles **b** in *Eudiaptomus gracilis* and *Daphnia longispina* fed with *Cryptomonas* sp. (C), *Dinobryon divergens* (D) and their 1:1 mixture (M) during the experimental period (21 days, *N* = 10 per treatment). Significant differences (*p* < 0.05) between treatments based on post hoc tests (Tukey HSD or Dunn’s test depending on the applied statistics) are indicated by letters. Note that in the case of cumulative number of juveniles in *E. gracilis*, significance is based on square root-transformed data but on the figure, we present the row data for an easier visualization of the original units. The detailed results of the applied statistics (Kruskal–Wallis test or one-way ANOVA) are presented in Supplementary Information, Table S2

**Fig. 4:**
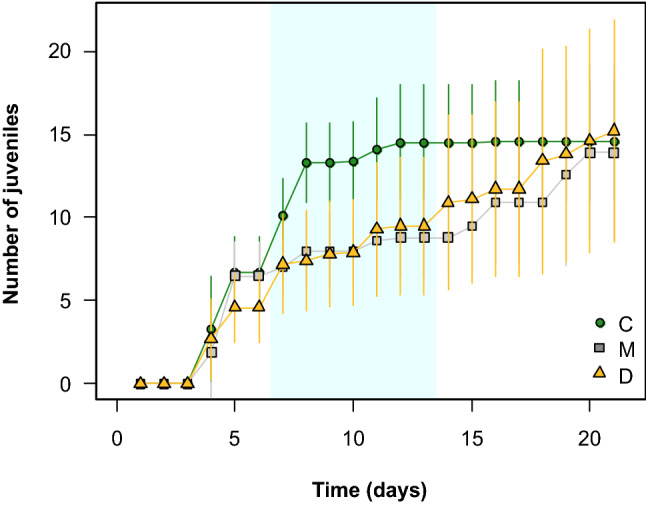
Cumulative number of juveniles per females (mean ± SD, *N* = 10 per treatment) in *Daphnia longispina* over the course of the experiment on the three food types (C—*Cryptomonas* sp., D—*Dinobryon divergens*, M—1:1 mixture). Coloured background indicates the time interval where number of juveniles in the *Cryptomonas* treatment significantly exceeded those on the other two (based on the results of one-way ANOVAs or Kruskal–Wallis tests, with post hoc tests for treatment-specific differences). Treatments with *D. divergens* and mixed food never differed significantly from each other

In the long-term feeding experiment, we found no significant treatment-specific differences in the survival of *E. gracilis* (Fig. 3a). However, we only recorded dead individuals in the *D. divergens* (*N* = 2 out of 10) and mixed treatments (*N* = 1). The cumulative number of juveniles per females was significantly different among the three diet types, being severely reduced on *D. divergens* (ca. nine times less juveniles compared to *Cryptomonas* considering the mean values) and being intermediate on the mixed diet (Fig. 3b). The results were similar in the case of the number of broods per females and mean brood size: both were significantly lower on *D. divergens* compared to *Cryptomonas* (Supplementary Information, Fig S2). In contrast to *E. gracilis*, we found significant treatment effects on *D. longispina* survival. Animals lived significantly longer on *D. divergens* compared to *Cryptomonas*, while the mixed diet had an intermediate effect (Fig. 3a). But this was not the case with offspring production with no significant differences among the treatments (Fig. 3b). However, the shorter time of survival in *D. longispina* coincided with faster reproduction on *Cryptomonas,* resulting in significantly more juveniles during the middle of the experiment compared to the other two treatments (which were never significantly different from each other). *D. longispina* feeding on *Cryptomonas* produced the same number of offspring in 8 days that was produced in 21 days by animals feeding on *D. divergens* and mixed diet (Fig. 4). This meant less broods but larger mean brood size on *Cryptomonas* (Supplementary Information, Fig S2).

**Fig. 5:**
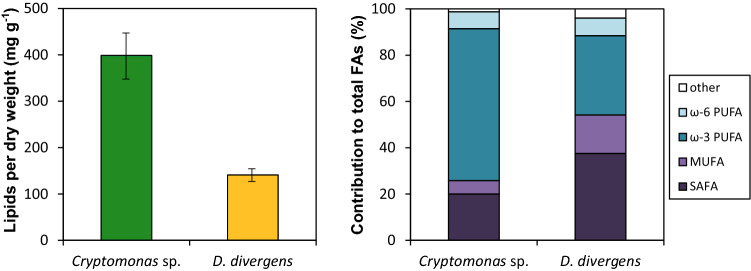
The total lipid content (left; mean ± SD calculated for technical replicates, *N* = 3) and fatty acid (FA) composition (right) of the two algal species used as food in the experiments. *PUFA* polyunsaturated fatty acids, *MUFA* monounsaturated fatty acids, *SAFA* saturated fatty acids

Biochemical analysis on the algal cultures revealed that *D. divergens* contains a low lipid content per dry weight, which is approx. only one third of that measured in *Cryptomonas* (Fig. 5). Consequently, the essential fatty acids contents were all lower in *D. divergens* (Supplementary Information, Table S3). Considering the relative amounts of different fatty acid groups, we found that *Cryptomonas* had a higher ratio of ω-3 fatty acids, while the percentages of ω-6 fatty acids were similar in the two algae. Elemental analysis revealed that *D. divergens* had higher (atomic) C:P (more than three times) and N:P ratio than *Cryptomonas* (Table 3).

**Table 3:** The atomic carbon:phosphorus (C:P) and nitrogen:phosphorus (N:P) ratio (mean ± SD stand for technical replicates) of the two algae

	C:P	N:P
*Dinobryon divergens*	504.5 ± 7.9	33.9 ± 0.9
*Cryptomonas* sp.	151.9 ± 6.2	21.5 ± 0.9

**Fig. 6:**
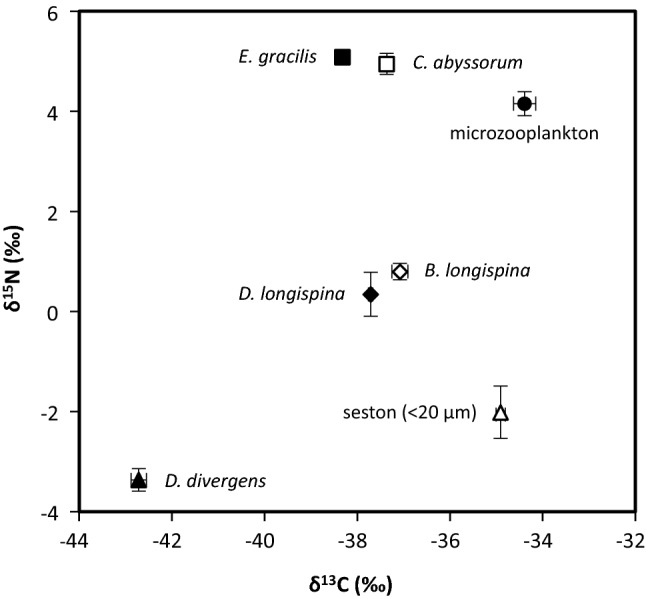
Stable isotope biplot (mean ± SD, *N* = 3) representing the trophic positions of planktonic groups or species in Lake Lunz

Analysis on plankton collected from Lake Lunz revealed more depleted δ^13^C values (mean ± SD: − 42.7 ± 0.2‰) in *D. divergens*, distinct from the small (< 20 µm) fraction of seston (− 34.9 ± 0.1‰; dominated by small chrysophytes, followed by synurophytes and cryptophytes) and zooplankton (mean values ranging from − 34.4 to − 38.3‰; Fig. 6). For example, the δ^13^C values of the primary consumer *D. longispina* (− 37.7 ± 0.06‰) were isotopically higher compared to the values that could have been reached on a *Dinobryon*-dominated diet based on the isotopically more depleted carbon values of *D. divergens* (− 42.4 to − 42.3‰). The δ^15^N values of *D. divergens* where somewhat lower (− 3.4 ± 0.2‰) than that of the small fraction of seston (− 2.0 ± 0.5‰), and clearly below the values obtained for zooplankton (mean values from 0.34 to 5.1‰; Fig. 6).

## Discussion

This study indicates that the chrysophyte *D. divergens*, which belongs to one of the most widespread and dominant mixotrophic flagellate genus in freshwaters, represents low-quality food and suppresses zooplankton reproduction. This is in clear contrast with recent research which suggests increased trophic transfer by mixotrophy (Ward and Follows 2016). Thus, results of the current study suggest that the typical traits of colonial mixotrophs diminish their contribution to secondary production.

The negative effect of *D. divergens* on zooplankton reproduction is likely a combined effect of grazing resistance and poor food quality (biochemical and elemental composition). Grazing resistance emerges from the loricas that can build up bushy colonies. It can be expected that the resulting reduced food uptake will be especially evident in *Daphnia* due to their feeding constraints as their filtration range is restricted to small particles and the avoidance of non-preferred particles is only possible by rejecting all food items collected on the filtering mesh (Sommer and Stibor 2002). This is confirmed in *D. longispina* by the steep decline in total ingested biomass and the over-proportional decrease in ingestion rates on *Cryptomonas* with increasing share of *D. divergens*. Finding that *D. longispina* ingested less *Cryptomonas* than expected based on its concentration in the medium clearly shows that the presence of *D. divergens* interferes with the uptake of high-quality food. Individual cells with their lorica coatings (average lorica length in our experiments was ~ 36 µm; Table 1) may already interfere with the filtering apparatus of *D. longispina* as they correspond to the upper particle size limit that can be ingested by similar-sized *Daphnia* species (Burns 1968). But colonies consisting of two (average length ~ 66 µm) or more cells are very likely to inhibit food uptake. The fact that the algae was provided on average in two-cell colonies (Table 2) implies that ingestion may be even more limited in natural plankton communities with much larger *Dinobryon* colonies (McKenrie et al. 1995; Sommer et al. 2001) and for smaller-sized cladocerans, such as *Bosmina* or *Ceriodaphnia* (Geller and Müller 1981). Given that with climate change, not only the size structure of zooplankton communities is expected to shift to smaller-sized species (Brucet et al. 2010), but also the body size of individual species is predicted to decrease with elevated temperature (Brans et al. 2017), this overall might lead to increasing dietary constraints for zooplankton and impeded energy transfer to higher trophic levels. In the copepod *E. gracilis*, biomass ingestion rates decreased more gradually from monospecific *Cryptomonas* to the pure *D. divergens* diet, which may be attributed to a higher ability to feed on *D. divergens* or preferential feeding on *Cryptomonas*. Our results provide stronger support for the latter. *E. gracilis* is able to ingest larger prey items such as rotifers (Šorf and Brandl 2012) and the *D. divergens* colonies used in the experiment were small, hence fell in the range that can be exploited by the copepod. Despite of this, *Eudiaptomus* did not ingest higher quantities of the chrysophyte (per total ingested biomass) than *D. longispina* in any of the three treatments with food mixtures. At the same time, the similar ingestion rates of *E. gracilis* on *Cryptomonas* in algae mixtures relative to those of the monospecific *Cryptomonas* indicate selective feeding on the naked flagellate.

Our results on the nutritional quality revealed high C:P ratios (~ 500) for *D. divergens* which is far from the proposed threshold ratio of 200–300 for *Daphnia* and therefore suggests P-limited growth and reproduction in the cladoceran (Hessen et al. 2013). Although less is known about such a threshold ratio in copepods, adults and late copepodid stages of calanoid copepods generally have higher body C:P and C:N ratios than cladocerans (Elser et al. 1996; Sommer and Stibor 2002; Meunier et al. 2016), suggesting that copepods would be less affected by higher dietary C:P ratios. The fact that *D. divergens* had a much stronger negative effect on the reproduction of *E. gracilis* compared to *D. longispina* therefore suggest other factors acting beyond food stoichiometry. One of these could be the essential fatty acid content of the food. However, we found that the contents of all lipids and essential fatty acids were lower in *D. divergens* than in *Cryptomonas* making it hard to stress any conclusions on the different sensitivity of the two zooplankton species. It is possible that other essential biomolecules (e.g., sterols, amino acids; Peltomaa et al. 2017) which were not targeted in our study also played a role leading to the observed differences.

Most likely a considerable fraction of carbon in *D. divergens* is allocated to the lorica consisting mostly of cellulose (Franke and Herth 1973), which cannot be assimilated efficiently by crustacean zooplankton (Schoenberg et al. 1984). We therefore argue that instead of a direct negative stoichiometric or biochemical effect, the main reason for the low food quality is rather that cellulose from the ingested loricas fills the digestive system with indigestible carbon, which interferes with the utilization of the more palatable food. Both the C:P ratio and the low amount of lipids per biomass in *D. divergens* may be somewhat overestimated in our study, as our culture also contained empty loricas which were included in the analysis together with those containing a cell. This is not an unnatural situation as empty loricas are typically found in *Dinobryon* colonies in nature depending on the stage of a bloom (S. Berger, pers. comm.). Feeding on *Dinobryon* therefore may result in starvation-like dietary responses in the consumers due to the high amount of ingested cellulose.

The observed grazing resistance and food quality suggest a generally negative effect on zooplankton life histories, which was evident in the long-term feeding experiments. Results on survival and reproduction furthermore reflected basic differences in life-history strategies and functional differences among cladocerans and copepods. Cladocerans evolved to fast growth and reproduction allowing them to rapidly exploit available resources and dominate over slower-growing copepods when population growth is not limited (Elser et al. 1996). Our results showed longevity but delayed reproductive output in *D. longispina* on *D. divergens*, which is a known response of the cladoceran to limited food quality or quantity (Vijverberg 1976; Becker and Boersma 2003), and imply reduced metabolic rates and population growth. Mixed diet with the high-quality food *Cryptomonas* did not result in higher reproductive output indicating that *D. longispina* could not compensate for the reduced food uptake likely caused by the mechanically interfering colonies. In the copepod *E. gracilis*, we did not find significant treatment effects on survival, but the few dead individuals and the overall weak condition of the animals (indicated by lower motility) towards the end of the experimental incubation indicated nutritional deficiency on *D. divergens*. It is likely that the negative effects on survival would become more evident on a time scale longer than our incubation period (21 days). But the effect on reproduction was dramatic, with an almost tenfold decrease in juveniles per female produced during the experiment. Providing *D. divergens* in mixture with the high-quality food resulted in significantly higher fitness, which complies with our observation on the grazing rates indicating that the copepod can at least partly meet its nutritional demand via its selective feeding. However, the fact that monospecific *Cryptomonas* still supported the highest reproductive output indicates that selective feeding has also an energetic cost.

Overall, while *Dinobryon* colonies may appear as an attractive ‘concentrated’ food source especially for copepods (Sommer et al. 2001), our results clearly show a generally low value in comparison with a naked unicellular cryptophyte. The negative dietary effects were especially evident in *E. gracilis* with dramatically reduced reproductive output on monospecific *D. divergens* diet. Our choice of *Cryptomonas* as reference food over other phytoplankton taxa that are widely used in zooplankton experiments (such as green algae for cladocerans) was based on the fact that it is considered among the few algal taxa of high nutritional value for freshwater calanoid copepods that are generally sensitive for unialgal diets (Hart and Santer 1994; von Elert and Stampfl 2000). Though we did not test this in the strain we used, there are numerous mixotrophic taxa within the genus *Cryptomonas* (e.g., Tranvik et al. 1989; Domaizon et al. 2003). The differences in the nutritional value of *Cryptomonas* and *D. divergens* suggests that there may be fundamental differences among mixotrophic taxa in their contribution to secondary production.

The results of the stable isotope analyses (based on trophic fractionation of carbon) provided further indication that *Dinobryon* is not a major food source for zooplankton in natural ecosystems. Its carbon signal was strongly depleted compared to other members of the plankton community in Lake Lunz, which may be linked to carbon uptake through bacterivory (Taipale et al. 2016). The δ^13^C value of the small-sized (< 20 µm) fraction of seston was more similar to those of zooplankton, which altogether indicated a stronger overall role of this fraction of seston as the basis for secondary production. This is in line with our experimental results on grazing resistance and avoidance. In natural systems, avoidance of *Dinobryon* can be further increased by spatial segregation. Especially in deeper lakes, vertical migration allows zooplankton to enhance their diet (Lampert et al. 2003), which may also contribute to such differences in carbon isotope signals between algae and grazers like in our study. However, climate-change driven increased stratification and DOC input will surely enhance resource monopolization by mixotrophs, including *Dinobryon*, extending their dominance over the season, which altogether means a decrease in the overall available food biomass for zooplankton. Looking at the nitrogen isotope signals, it may seem surprising that the mixotrophic *D. divergens* had a lower δ^15^N value than seston. This may be due to the fact that dissolved nitrogen is generally non-limiting in Lake Lunz, and therefore even mixotrophic algae may meet their nitrogen requirement by utilizing dissolved nitrogen. Furthermore, our bulk seston samples likely contained small herbivores such as ciliates and rotifers which altogether may lift the δ^15^N signal.

Global climate change is expected to increase the occurrence and dominance of chrysophyte algae in oligo- and mesotrophic lakes. *Dinobryon*, one of their most common representatives, was repeatedly found to become increasingly dominant as a response to DOC-enrichment (Bell et al. 1993; Urrutia‐Cordero et al. 2017) and oligotrophication (Dokulil and Teubner 2005; Kamjunke et al. 2007). Here we provided evidence that it represents low-quality food for zooplankton and therefore its dominance may have serious consequences for pelagic carbon flow by altering secondary production with possible species-specific differences in consumers. Our findings, together with deleterious effects on zooplankton reported for other species of chrysophytes (Boenigk and Stadler 2004; Hiltunen et al. 2012), highlight the need for considering taxonomic differences (e.g., cryptophytes are high-quality food) and taxon-specific traits (e.g., grazing resistance, toxins) when assessing the food quality of mixotrophic protists. Such information is critical to resolve uncertainties and refine global models on the importance of mixotrophy in trophic webs.

## Electronic supplementary material

Below is the link to the electronic supplementary material.

Supplementary file1 (PDF 212 kb)

## Data Availability

The datasets generated during and/or analysed during the current study are available from the corresponding author on reasonable request.

## References

[CR1] Adrian R, Schneider-Olt B (1999). Top-down effects of crustacean zooplankton on pelagic microorganisms in a mesotrophic lake. J Plankton Res.

[CR2] Adrian R, O’Reilly CM, Zagarese H (2009). Lakes as sentinels of climate change. Limnol Oceanogr.

[CR3] Agrawal AA (1998). Algal defense, grazers, and their interactions in aquatic trophic cascades. Acta Oecologica.

[CR4] Ahlgren G, Lundstedt L, Brett M, Forsberg C (1990). Lipid composition and food quality of some freshwater phytoplankton for cladoceran zooplankters. J Plankton Res.

[CR5] Atkinson A (1995). Omnivory and feeding selectivity in five copepod species during spring in the Bellingshausen Sea, Antarctica. ICES J Mar Sci.

[CR6] Becker C, Boersma M (2003). Resource quality effects on life histories of *Daphnia*. Limnol Oceanogr.

[CR7] Bell RT, Vrede K, Stensdotter-Blomberg U, Blomqvist P (1993). Stimulation of the microbial food web in an oligotrophic, slightly acidified lake. Limnol Oceanogr.

[CR8] Berger I, Maier G (2001). The mating and reproductive biology of the freshwater planktonic calanoid copepod *Eudiaptomus gracilis*. Freshw Biol.

[CR9] Bergström A-K, Jansson M, Drakare S, Blomqvist P (2003). Occurrence of mixotrophic flagellates in relation to bacterioplankton production, light regime and availability of inorganic nutrients in unproductive lakes with differing humic contents. Freshw Biol.

[CR10] Bertoni R, Callieri C, Corno G (2002). The mixotrophic flagellates as key organisms from DOC to *Daphnia* in an oligotrophic alpine lake. Int Ver Theor Angew Limnol Verhandlungen.

[CR11] Bird DF, Kalff J (1986). Bacterial grazing by planktonic lake algae. Science.

[CR12] Boenigk J, Stadler P (2004). Potential toxicity of chrysophytes affiliated with Poterioochromonas and related ‘Spumella-like’flagellates. J Plankton Res.

[CR13] Branco P, Egas M, Elser JJ, Huisman J (2018). Eco-evolutionary dynamics of ecological stoichiometry in plankton communities. Am Nat.

[CR14] Brandl Z (2005). Freshwater copepods and rotifers: predators and their prey. Hydrobiologia.

[CR15] Brans KI, Jansen M, Vanoverbeke J (2017). The heat is on: genetic adaptation to urbanization mediated by thermal tolerance and body size. Glob Change Biol.

[CR16] Brucet S, Boix D, Quintana XD (2010). Factors influencing zooplankton size structure at contrasting temperatures in coastal shallow lakes: implications for effects of climate change. Limnol Oceanogr.

[CR17] Burns CW (1968). The relationship between body size of filter-feeding cladocera and the maximum size of particle ingested. Limnol Oceanogr.

[CR18] Cabrerizo MJ, Álvarez-Manzaneda MI, León-Palmero E (2020). Warming and CO_2_ effects under oligotrophication on temperate phytoplankton communities. Water Res.

[CR19] Callieri C, Bertoni R, Contesini M, Bertoni F (2014). Lake level fluctuations boost toxic cyanobacterial “Oligotrophic Blooms”. PLoS ONE.

[CR20] Clesceri LS, Greenberg AE, Eaton AD (1999). Standard methods for the examination of water and wastewater.

[CR21] Colina M, Calliari D, Carballo C, Kruk C (2016). A trait-based approach to summarize zooplankton–phytoplankton interactions in freshwaters. Hydrobiologia.

[CR22] de Wit HA, Valinia S, Weyhenmeyer GA (2016). Current browning of surface waters will be further promoted by wetter climate. Environ Sci Technol Lett.

[CR23] DeMott WR (1986). The role of taste in food selection by freshwater zooplankton. Oecologia.

[CR24] Dokulil MT, Skolaut C (1991). Aspects of phytoplankton seasonal succession in Mondsee, Austria, with particular reference to the ecology of *Dinobryon* EHRENB. Verhandlungen Int Ver Für Theor Angew Limnol.

[CR25] Dokulil MT, Teubner K (2005). Do phytoplankton communities correctly track trophic changes? An assessment using directly measured and palaeolimnological data. Freshw Biol.

[CR26] Domaizon I, Viboud S, Fontvieille D (2003). Taxon-specific and seasonal variations in flagellates grazing on heterotrophic bacteria in the oligotrophic Lake Annecy—importance of mixotrophy. FEMS Microbiol Ecol.

[CR27] Elser JJ, Dobberfuhl DR, MacKay NA, Schampel JH (1996). Organism size, life history, and N:P StoichiometryToward a unified view of cellular and ecosystem processes. Bioscience.

[CR28] Faithfull CL, Wenzel A, Vrede T, Bergström A-K (2011). Testing the light: nutrient hypothesis in an oligotrophic boreal lake. Ecosphere.

[CR29] Fischer R, Giebel H-A, Hillebrand H, Ptacnik R (2017). Importance of mixotrophic bacterivory can be predicted by light and loss rates. Oikos.

[CR30] Flynn KJ, Mitra A, Glibert PM, Burkholder JM, Glibert PM, Berdalet E, Burford MA (2018). Mixotrophy in harmful algal blooms: by whom, on whom, when, why, and what next. Global ecology and oceanography of harmful algal blooms.

[CR31] Forsström L, Roiha T, Rautio M (2013). Responses of microbial food web to increased allochthonous DOM in an oligotrophic subarctic lake. Aquat Microb Ecol.

[CR32] Franke WW, Herth W (1973). Cell and lorica fine structure of the chrysomonad alga, *Dinobryon sertularia* Ehr. (Chrysophyceae). Arch Für Mikrobiol.

[CR33] Frost BW (1972). Effects of size and concentration of food particles on the feeding behavior of the marine planktonic copepod Calanus pacificus. Limnol Oceanogr.

[CR34] Fussmann G (1996). The importance of crustacean zooplankton in structuring rotifer and phytoplankton communities; an enclosure study. J Plankton Res.

[CR35] Geller W, Müller H (1981). The filtration apparatus of cladocera: filter mesh-sizes and their implications on food selectivity. Oecologia.

[CR36] Grey J, Jones RI (1999). Carbon stable isotopes reveal complex trophic interactions in lake plankton. Rapid Commun Mass Spectrom.

[CR37] Hansen HP, Koroleff F, Grasshoff K, Kremling K, Ehrhardt M (2007). Determination of nutrients. Methods of seawater analysis.

[CR38] Hart RC, Santer B (1994). Nutritional suitability of some uni-algal diets for freshwater calanoids: unexpected inadequacies of commonly used edible greens and others. Freshw Biol.

[CR39] Hartmann M, Grob C, Tarran GA (2012). Mixotrophic basis of Atlantic oligotrophic ecosystems. Proc Natl Acad Sci USA.

[CR40] Heissenberger M, Watzke J, Kainz MJ (2010). Effect of nutrition on fatty acid profiles of riverine, lacustrine, and aquaculture-raised salmonids of pre-alpine habitats. Hydrobiologia.

[CR41] Hessen DO (1992). Nutrient element limitation of zooplankton production. Am Nat.

[CR42] Hessen DO, Elser JJ, Sterner RW, Urabe J (2013). Ecological stoichiometry: an elementary approach using basic principles. Limnol Oceanogr.

[CR43] Hiltunen T, Barreiro A, Hairston NG (2012). Mixotrophy and the toxicity of Ochromonas in pelagic food webs. Freshw Biol.

[CR44] Huisman J, Codd GA, Paerl HW (2018). Cyanobacterial blooms. Nat Rev Microbiol.

[CR45] Infante A (1973). Untersuchungen über die Ausnutzbarkeit verschiedener Algen durch das zooplankton. Arch Für Hydrobiol Suppl.

[CR46] Jäger CG, Vrede T, Persson L, Jansson M (2014). Interactions between metazoans, autotrophs, mixotrophs and bacterioplankton in nutrient-depleted high DOC environments: a long-term experiment. Freshw Biol.

[CR47] Jeppesen E, Søndergaard M, Jensen JP (2005). Lake responses to reduced nutrient loading—an analysis of contemporary long-term data from 35 case studies. Freshw Biol.

[CR48] Kainz MJ, Ptacnik R, Rasconi S, Hager HH (2017). Irregular changes in lake surface water temperature and ice cover in subalpine Lake Lunz, Austria. Inland Waters.

[CR49] Kamjunke N, Henrichs T, Gaedke U (2007). Phosphorus gain by bacterivory promotes the mixotrophic flagellate *Dinobryon* spp. during re-oligotrophication. J Plankton Res.

[CR50] Katechakis A, Stibor H (2006). The mixotroph Ochromonas tuberculata may invade and suppress specialist phago- and phototroph plankton communities depending on nutrient conditions. Oecologia.

[CR51] Katechakis A, Haseneder T, Kling R, Stibor H (2005). Mixotrophic versus photoautotrophic specialist algae as food for zooplankton: the light: nutrient hypothesis might not hold for mixotrophs. Limnol Oceanogr.

[CR52] Kerfoot WC, Kirk KL (1991). Degree of taste discrimination among suspension-feeding cladocerans and copepods: Implications for detritivory and herbivory. Limnol Oceanogr.

[CR53] Kies L (1967). Über Zellteilung und Zygotenbildung bei Roya obtusa (Breb.) West et West. Mitteilungen Staatsinst Für Allg Bot Hambg.

[CR54] Kiørboe T (2011). How zooplankton feed: mechanisms, traits and trade-offs. Biol Rev.

[CR55] Kleppel GS (1993). On the diets of calanoid copepods. Mar Ecol Prog Ser.

[CR56] Knisely K, Geller W (1986). Selective feeding of four zooplankton species on natural lake phytoplankton. Oecologia.

[CR57] Lampert W, McCauley E, Manly BFJ (2003). Trade-offs in the vertical distribution of zooplankton: ideal free distribution with costs?. Proc R Soc Lond B Biol Sci.

[CR58] Lehman JT (1976). Ecological and nutritional studies on *Dinobryon Ehrenb*.: seasonal periodicity and the phosphate toxicity problem. Limnol Oceanogr.

[CR59] McCutchan JH, Lewis WM, Kendall C, McGrath CC (2003). Variation in trophic shift for stable isotope ratios of carbon, nitrogen, and sulfur. Oikos.

[CR60] McKenrie CH, Deibel D, Paranjape MA, Thompson RJ (1995). The marine mixotroph *Dinobryon Balticum* (chrysophyceae): phagotrophy and survival in a cold ocean1. J Phycol.

[CR61] Meunier CL, Boersma M, Wiltshire KH, Malzahn AM (2016). Zooplankton eat what they need: copepod selective feeding and potential consequences for marine systems. Oikos.

[CR62] Moorthi SD, Ptacnik R, Sanders RW (2017). The functional role of planktonic mixotrophs in altering seston stoichiometry. Aquat Microb Ecol.

[CR63] Müller-Navarra D, Lampert W (1996). Seasonal patterns of food limitation in *Daphnia galeata*: separating food quantity and food quality effects. J Plankton Res.

[CR64] Nejstgaard JC, Naustvoll L-J, Sazhin A (2001). Correcting for underestimation of microzooplankton grazing in bottle incubation experiments with mesozooplankton. Mar Ecol Prog Ser.

[CR65] O’Neil JM, Davis TW, Burford MA, Gobler CJ (2012). The rise of harmful cyanobacteria blooms: the potential roles of eutrophication and climate change. Harmful Algae.

[CR66] Peltomaa ET, Aalto SL, Vuorio KM, Taipale SJ (2017). The importance of phytoplankton biomolecule availability for secondary production. Front Ecol Evol.

[CR67] Persson J, Vrede T (2006). Polyunsaturated fatty acids in zooplankton: variation due to taxonomy and trophic position. Freshw Biol.

[CR68] Post DM (2002). Using stable isotopes to estimate trophic position: models, methods, and assumptions. Ecology.

[CR69] Ptacnik R, Lepistö L, Willén E (2008). Quantitative responses of lake phytoplankton to eutrophication in Northern Europe. Aquat Ecol.

[CR70] Ptacnik R, Gomes A, Royer S-J (2016). A light-induced shortcut in the planktonic microbial loop. Sci Rep.

[CR71] Pugnetti A, Bettinetti R (1999). Biomass and species structure of the phytoplankton of an high mountain lake (Lake Paione Superiore, Central Alps, Italy). J Limnol.

[CR72] Reynolds CS, Huszar V, Kruk C (2002). Towards a functional classification of the freshwater phytoplankton. J Plankton Res.

[CR73] Rocha O, Duncan A (1985). The relationship between cell carbon and cell volume in freshwater algal species used in zooplanktonic studies. J Plankton Res.

[CR74] Roulet N, Moore TR (2006). Environmental chemistry: browning the waters. Nature.

[CR75] Sanders RW, Porter KG, Bennett SJ, DeBiase AE (1989). Seasonal patterns of bacterivory by flagellates, ciliates, rotifers, and cladocerans in a freshwater planktonic community. Limnol Oceanogr.

[CR76] Sandgren CD, Sandgren CD (1988). The ecology of chrysophyte flagellates: their growth and perennation strategies as freshwater phytoplankton. Growth and reproductive strategies of freshwater phytoplankton.

[CR77] Schoenberg SA, Maccubbin AE, Hodson RE (1984). Cellulose digestion by freshwater microcrustacea. Limnol Oceanogr.

[CR78] Smyntek PM, Teece MA, Schulz KL, Storch AJ (2008). Taxonomic differences in the essential fatty acid composition of groups of freshwater zooplankton relate to reproductive demands and generation time. Freshw Biol.

[CR79] Sommer U, Sommer F (2006). Cladocerans versus copepods: the cause of contrasting top–down controls on freshwater and marine phytoplankton. Oecologia.

[CR80] Sommer U, Stibor H (2002). Copepoda—cladocera—tunicata: the role of three major mesozooplankton groups in pelagic food webs. Ecol Res.

[CR81] Sommer U, Sommer F, Santer B (2001). Complementary impact of copepods and cladocerans on phytoplankton. Ecol Lett.

[CR82] Sommer U, Sommer F, Santer B (2003). Daphnia versus copepod impact on summer phytoplankton: functional compensation at both trophic levels. Oecologia.

[CR83] Šorf M, Brandl Z (2012). The rotifer contribution to the diet of *Eudiaptomus gracilis* (G. O. Sars, 1863) (copepoda, calanoida). Crustaceana.

[CR84] Svensson J-E, Stenson JAE (1991). Herbivoran impact on phytoplankton community structure. Hydrobiologia.

[CR85] Taipale SJ, Vuorio K, Brett MT (2016). Lake zooplankton δ^13^C values are strongly correlated with the δ^13^C values of distinct phytoplankton taxa. Ecosphere.

[CR86] Talling JF (2003). Phytoplankton–zooplankton seasonal timing and the ‘clear-water phase’ in some English lakes. Freshw Biol.

[CR87] Taranu ZE, Gregory-Eaves I, Leavitt PR (2015). Acceleration of cyanobacterial dominance in north temperate-subarctic lakes during the Anthropocene. Ecol Lett.

[CR88] Tong Y, Zhang W, Wang X (2017). Decline in Chinese lake phosphorus concentration accompanied by shift in sources since 2006. Nat Geosci.

[CR89] Tranvik LJ, Porter KG, Sieburth JMcN (1989). Occurrence of bacterivory in Cryptomonas, a common freshwater phytoplankter. Oecologia.

[CR90] Urrutia-Cordero P, Ekvall MK, Ratcovich J (2017). Phytoplankton diversity loss along a gradient of future warming and brownification in freshwater mesocosms. Freshw Biol.

[CR91] Verbeek L, Gall A, Hillebrand H, Striebel M (2018). Warming and oligotrophication cause shifts in freshwater phytoplankton communities. Glob Change Biol.

[CR92] Vijverberg J (1976). The effect of food quantity and quality on the growth, birth-rate and longevity of *Daphnia hyalina Leydig*. Hydrobiologia.

[CR93] von Elert E, Stampfl P (2000). Food quality for Eudiaptomus gracilis: the importance of particular highly unsaturated fatty acids. Freshw Biol.

[CR94] Ward BA, Follows MJ (2016). Marine mixotrophy increases trophic transfer efficiency, mean organism size, and vertical carbon flux. Proc Natl Acad Sci.

[CR95] Watson SB, McCauley E, Downing JA (1997). Patterns in phytoplankton taxonomic composition across temperate lakes of differing nutrient status. Limnol Oceanogr.

[CR96] Watson SB, Whitton BA, Higgins SN, Wehr JD, Sheath RG, Kociolek JP (2015). Chapter 20—harmful algal blooms. Freshwater Algae of North America.

[CR97] Wilken S, Soares M, Urrutia-Cordero P (2018). Primary producers or consumers? Increasing phytoplankton bacterivory along a gradient of lake warming and browning. Limnol Oceanogr.

[CR98] Winder M, Reuter JE, Schladow SG (2009). Lake warming favours small-sized planktonic diatom species. Proc R Soc B Biol Sci.

[CR99] Wood SN (2017). Generalized additive models: an introduction with R.

[CR100] Zubkov MV, Tarran GA (2008). High bacterivory by the smallest phytoplankton in the North Atlantic Ocean. Nature.

