# Supplementary material for: Grazing resistance and poor food quality of a widespread mixotroph impair zooplankton secondary production
Source: Oecologia. 2020 Jun 5;193(2):489–502. doi: 10.1007/s00442-020-04677-x (PMC7320944; doi:10.1007/s00442-020-04677-x)
Supplement: Supplementary file 1 — Supplementary file1 (PDF 212 kb) [file 442_2020_4677_MOESM1_ESM.pdf]

Grazing resistance and poor food quality of a widespread mixotroph impair zooplankton secondary production

Csaba F. Vad[*], C. Schneider, Dunja Lukić, Zsófia Horváth, Martin J. Kainz, Herwig Stibor, Robert Ptacnik

corresponding author: vad.csaba@gmail.com, WasserCluster Lunz, Dr. Carl Kupelwieser Promenade 5, A-3293 Lunz am See, Austria & Balaton Limnological Institute, Centre for Ecological Research, Klebelsberg Kuno u. 3, H-8237, Tihany, Hungary

Table S1 – Results of Kruskal-Wallis tests and one-way ANOVAs testing for differences in the life history parameters recorded in the long-term experiment on the three different food items.

		K-W test			one-way ANOVA		
		df	*chi*^{2}	*p*	df	*F*	*p*
**Survival**	*E. gracilis*	2	2.14	0.343			
	D. longispina	2	10.31	<0.01			
**No. of juveniles per female**	*E. gracilis*				2, 29	20.41	<0.001
	D. longispina				2, 29	0.148	0.863
**No. of broods per female**	*E. gracilis*				2, 29	14.14	<0.001
	D. longispina	2	7.16	<0.05			
**Mean brood size (no. of juveniles per brood)**	*E. gracilis*				2, 29	13.03	<0.001
	D. longispina				2, 29	9.30	<0.001

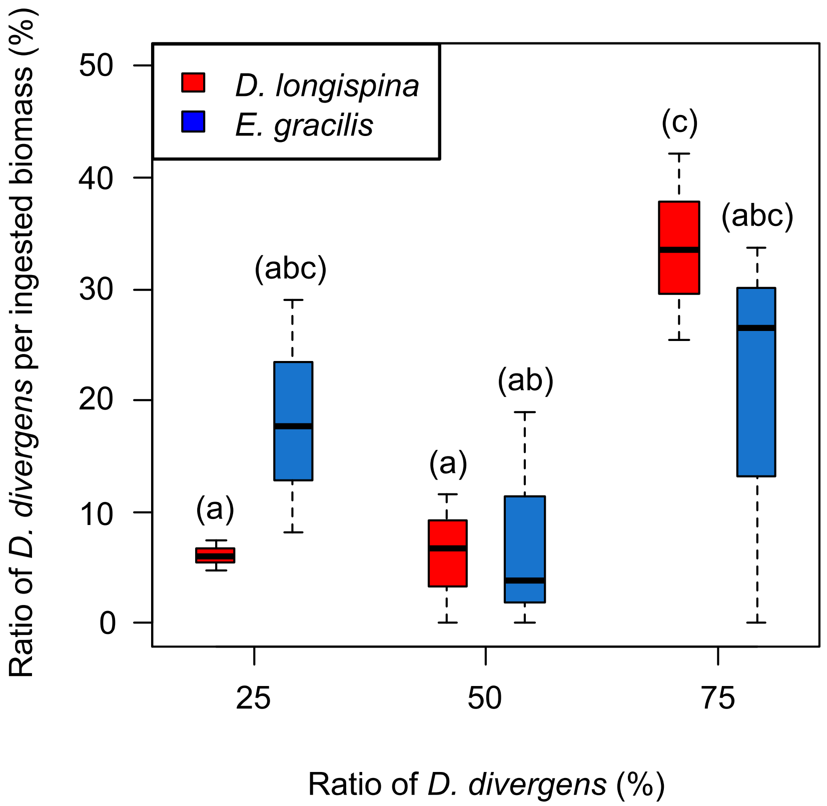

Figure S1 – The ratio of ingested *Dinobryon divergens* per total ingested biomass across treatments. Labels in brackets indicate marginally significant differences (p values spread between 0.055 and 0.074) among treatment combinations resulting from Tukey HSD tests. Results of two-way ANOVA for the data presented here are listed in Table S3.

Table S2 - Results of two-way ANOVA testing for differences in the ratio of ingested *Dinobyon divergens* in the total ingested biomass among treatments and species. Differences among groups of categorical variables were tested by Tukey's HSD *post hoc* tests: '=' stands for non-significant, '<' for significant ($p < 0.05$), '(<)' for marginally significant ($p < 0.1$) differences.

	df	*F*	*p*	*post hoc* comparisons
treatment	1	6.15	< 0.05	25% = 50%, 25% (<) 75%, 50% < 75%
species	2	0.0	0.99	no test was performed
treatment*species	2	2.40	0.13	presented on Fig S1

Table S3 – Absolute (mean ± SD stand for technical replicates) and relative amounts of individual fatty acids (FA) in the two algae used as food in the experiments. Abbrev.: MUFA – monounsaturated fatty acids, PUFA – polyunsaturated fatty acids, SAFA – saturated fatty acids. Essential fatty acids (following the terminology of Kainz et al. 2004) are highlighted in bold.

	Mass fractions (μg mg dry weight^{-1})		Relative content (% of total FAs)	
Fatty acid	*Cryptomonas* sp.	*D. divergens*	*Cryptomonas* sp.	*D. divergens*
SAFA				
14:0	2.31 ± 0.05	3.01 ± 0.13	3.02 ± 0.1	23.37 ± 1.51
15:0	0.23 ± 0.08	0.02 ± 0.00	0.30 ± 0.1	0.14 ± 0.01
16:0	10.21 ± 0.37	1.17 ± 0.02	13.31 ± 0.36	9.09 ± 0.38
17:0	0.37 ± 0.02	0.01 ± 0.02	0.49 ± 0.04	0.10 ± 0.13
18:0	2.17 ± 0.47	0.59 ± 0.07	2.86 ± 0.77	4.60 ± 0.38
20:0	0.05 ± 0.02	0.03 ± 0.00	0.06 ± 0.03	0.27 ± 0.13
MUFA				
16.1ω9	0.24 ± 0.07	0.10 ± 0.01	0.31 ± 0.08	0.78 ± 0.02
16.1ω7	1.06 ± 0.06	0.86 ± 0.07	1.38 ± 0.01	6.69 ± 0.42
18.1ω7	1.21 ± 0.11	0.49 ± 0.02	1.57 ± 0.09	3.81 ± 0.09
18.1ω9t	0.26 ± 0.03	0.00 ± 0.00	0.33 ± 0.02	0.00 ± 0.00
18.1ω9c	1.68 ± 0.11	0.67 ± 0.10	2.19 ± 0.08	5.14 ± 0.66
18.1ω12	0.00 ± 0.00	0.02 ± 0.03	0.00 ± 0.00	0.17 ±0.25
PUFA				
**18.2ω6**	**5.38 ± 0.33**	**0.67 ± 0.03**	**6.99 ± 0.06**	**5.20 ± 0.15**
**18.3ω3**	**16.67 ± 1.26**	**0.24 ± 0.01**	**21.67 ± 0.48**	**1.89 ± 0.07**
18.3ω6	0.18 ± 0.04	0.17 ± 0.02	0.24 ± 0.07	1.34 ± 0.14
18.4ω3	16.95 ± 1.23	2.95 ± 0.11	22.04 ± 0.35	22.83 ± 0.20
20.3ω6	0.00 ± 0.000	0.09 ± 0.01	0.00 ± 0.00	0.72 ± 0.08
**20.4ω6**	**0.09 ± 0.01**	**0.05 ± 0.03**	**0.12 ± 0.01**	**0.36 ± 0.26**
**20.5ω3**	**15.05 ± 1.13**	**0.46 ± 0.02**	**19.56 ± 0.35**	**3.55 ± 0.05**
22.3ω3	0.00 ± 0.0	0.12 ± 0.01	0.00 ± 0.00	0.95 ± 0.05
22.5ω3	0.14 ± 0.01	0.10 ± 0.01	0.18 ± 0.01	0.80 ± 0.03
**22.6ω3**	**1.67 ± 0.12**	**0.55 ± 0.03**	**2.17 ± 0.04**	**4.28 ± 0.07**

Reference:

Kainz, M., Arts, M. T., & Mazumder, A. (2004). Essential fatty acids in the planktonic food web and their ecological role for higher trophic levels. *Limnology and Oceanography 49*(5), 1784–1793. https://doi.org/10.4319/lo.2004.49.5.1784

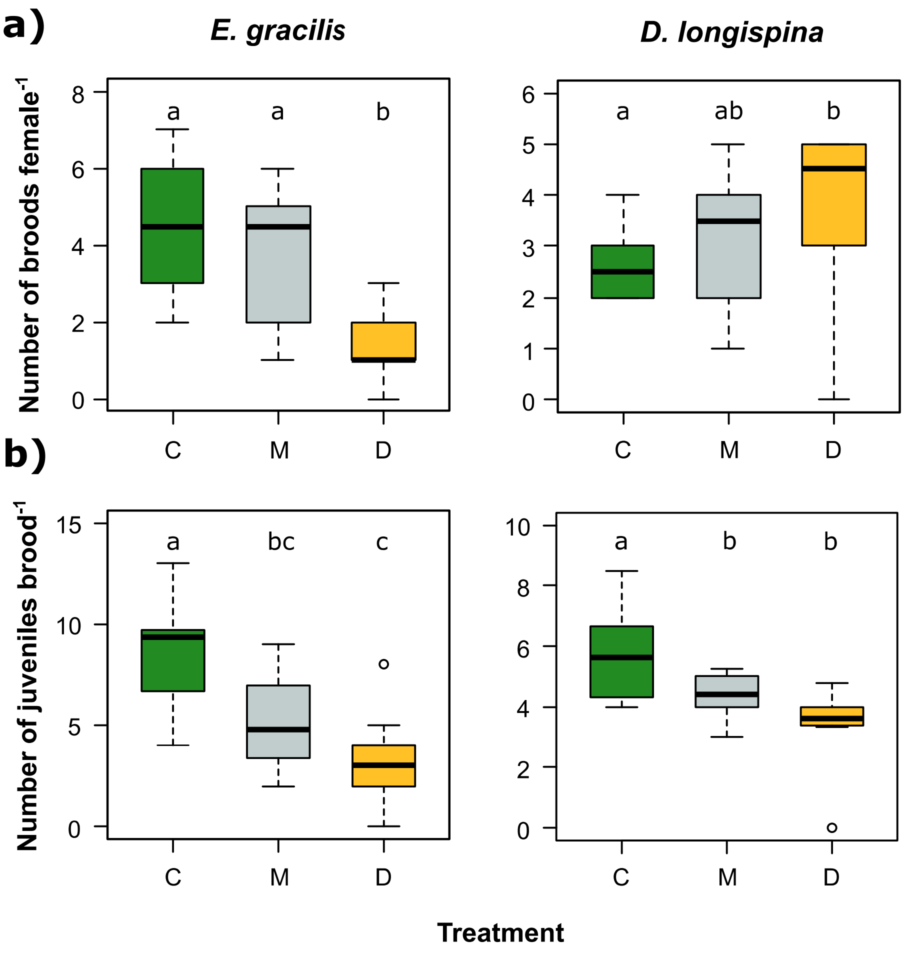

Fig. S2 – The mean number of broods (a) and mean brood size (b) in *Eudiaptomus gracilis* and *Daphnia longispina* fed with *Cryptomonas* (C), *D. divergens* (D) and their 1:1 mixture (M) during the experimental period (21 days). Significant differences ($p < 0.05$) between treatments based on *post hoc* tests (Tukey HSD or Dunn's test depending on the applied statistics) are indicated by letters. The detailed results of the applied statistics (Kruskal-Wallis test or one-way ANOVA) are presented in Supporting information, Table S2.
